# Recruitment Efforts for the study “Development and Validation of TeleNeuropsychology Normative Data for Older Adults”

**DOI:** 10.1002/alz.092432

**Published:** 2025-01-03

**Authors:** Ambar R Perez‐Lao, Gelan Ying, David E Marra, Lauren Santos, Franchesca Arias, Glenn E. Smith

**Affiliations:** ^1^ University of Florida, Gainesville, FL USA; ^2^ VA Boston Healthcare System, Boston, MA USA

## Abstract

**Background:**

Remote administration of well‐established neuropsychological instrument**s (**TeleNeuropsychological or TeleNP) can reduce assessment wait times and expand access to cognitive assessments for medically compromised and socially disadvantaged patients. A major limitation in the widespread uptake of TeleNP relates to the need for more normative data compared to in‐person assessments. This presentation describes a novel ascertainment strategy used in Florida’s Older Adults TeleNeuropsychology (FLOAT) project to identify and recruit “cognitively healthy” older adults to norm well‐established neuropsychological instruments administered remotely. This abstract summarizes recruitment efforts during Phase I and II of this study.

**Method:**

We utilize a previously developed machine learning algorithm to identify participants aged 50+ with a low‐to‐high probability of developing Alzheimer’s disease and related dementias (ADRD). We select those with low probabilities for invitation to participate. Prospective participants with severe psychiatric disorders (i.e., schizophrenia, bipolar disorder) or no internet access are excluded. About 7000 prospective participants between 51 to 104 years of age from the OneFlorida Data Repository were identified. Phases I and II used email to reach participants ages 60‐79 with the lowest ADRD risk.

**Result:**

Of the 5112 persons eligible for Phases I and II, 606 participants did not include emails in their contact information. Of those contacted, 4,347 did not respond to the first outreach contact, 55 declined to participate, and three did not meet the study criteria. Overall, 107 agreed to participate, and 30 are currently enrolled. As recruitment waves continue, these numbers will include participants aged 50‐59 and 80+.

**Conclusion:**

FLOAT incorporates machine‐learning‐based informatics to efficiently identify a robust sample of cognitively healthy older adults to norm a TeleNP protocol. Our initial affirmative response rate (2.4%) is promising. Additional strategies to improve response and enrollment rates are underway. New methods to leverage the algorithm while enriching the cohort for underrepresented groups are being explored and discussed, and preliminary normative data will be presented. Recruiting older adults for norm development is resource‐intensive and requires efficient yet flexible methods.

